# Supplementary material for: MEIOC prevents continued mitotic cycling and promotes meiotic entry during mouse oogenesis
Source: bioRxiv. 2025 Jun 15:2025.06.12.659330. Preprint. [Version 1] doi: 10.1101/2025.06.12.659330 (PMC12258517; doi:10.1101/2025.06.12.659330)
Supplement: 1 [file NIHPP2025.06.12.659330V1-supplement-1.pdf]

bioRxiv preprint doi: https://doi.org/10.1101/2025.06.12.659330; this version posted June 15, 2025. The copyright holder for this preprint (which was not certified by peer review) is the author/funder, who has granted bioRxiv a license to display the preprint in perpetuity. It is made available under aCC-BY 4.0 International license.

Figure S1

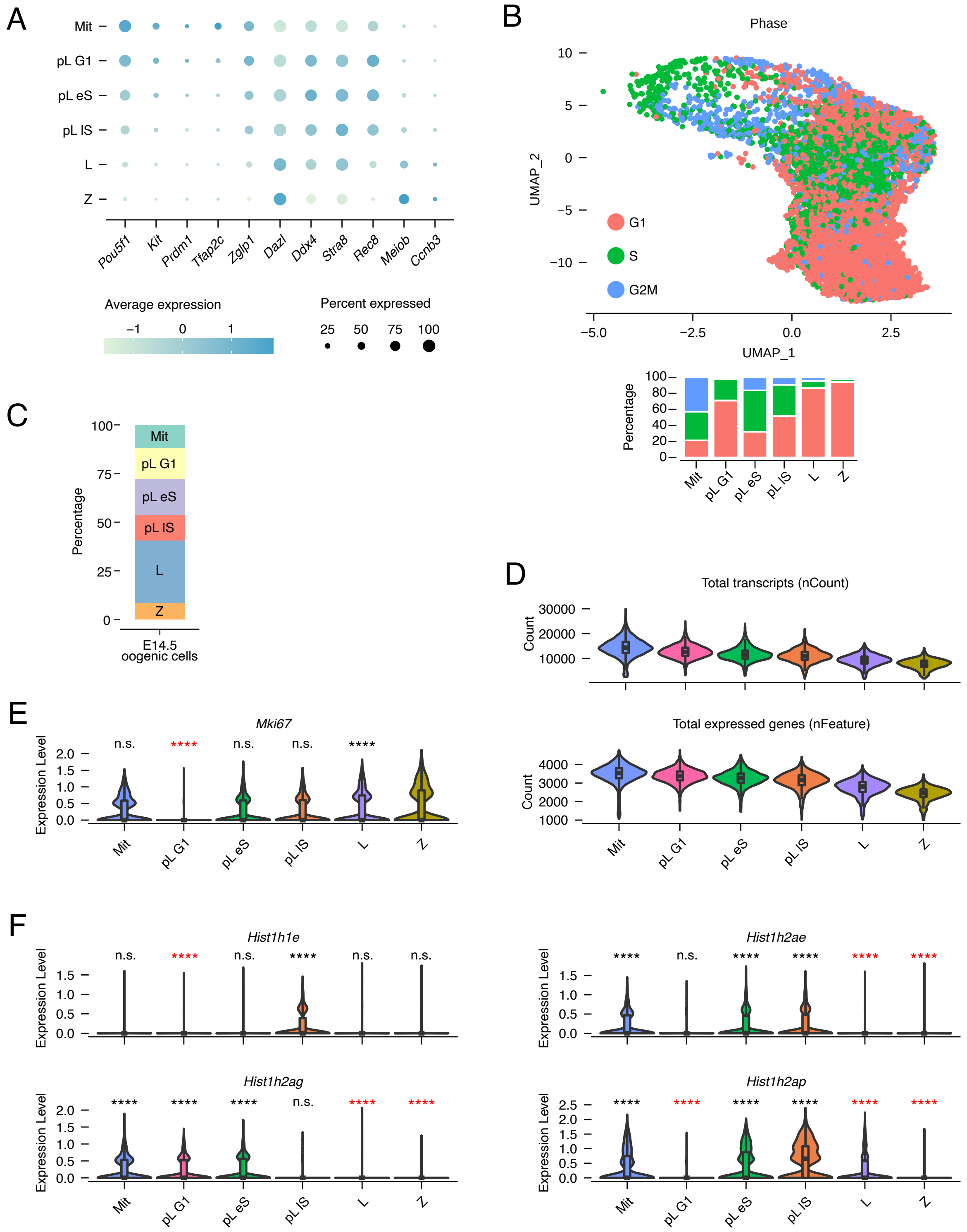

bioRxiv preprint doi: https://doi.org/10.1101/2025.06.12.659330; this version posted June 15, 2025. The copyright holder for this preprint (which was not certified by peer review) is the author/funder, who has granted bioRxiv a license to display the preprint in perpetuity. It is made available under aCC-BY 4.0 International license.

Figure S1. Identification of E14.5 oogenic cell subpopulations across the mitosis-to-meiosis transition via scRNA-seq.

(A) Dotplot of expression levels and percentage of cells for markers used to assign clusters. Data from scRNA-seq of *Pou5f1*:EGFP-positive cells from E14.5 ovary (Zhao et al., 2020).

(B) UMAP and per-cluster barplot of Seurat-based cell cycle phase designations used to assign clusters.

(C) Percentage of each oogenic cell type identified, relative to all oogenic cells.

(D) Total number of transcripts detected (nCount) and genes expressed (nFeature) in oogenic cell clusters.

(E) pL G1 cluster exhibits depleted *Mki67*, which is at its lowest during G1 phase of the cell cycle and thereby supports the cluster's G1 phase designation.

(F) pL eS and lS clusters exhibit enrichment of replication-dependent histones *Hist1h1e*, *Hist1h2ae*, *Hist1h2ag*, and *Hist1h2ap*, which confirms the clusters' S phase designation.

****, adj. P<0.0001; n.s., not significant. Red and black asterisks mark statistical depletion and enrichment, respectively.
